# Spatial and temporal variation of benthic macroinvertebrate communities along an urban river in Greater Manchester, UK

**DOI:** 10.1007/s10661-019-8019-6

**Published:** 2020-01-03

**Authors:** Cecilia Medupin

**Affiliations:** School of Earth and Environmental Sciences, The University of Manchester, Oxford Road, Manchester, M13 9PL UK

**Keywords:** Benthic macroinvertebrates, Physicochemical, Biotic indices, Multivariate tests, water quality, urban area

## Abstract

Urban rivers face challenges of increased human activities which also affect river organisms. In order to enhance freshwater biodiversity in urban rivers, it is important to determine how the benthic macroinvertebrate communities are influenced by key abiotic factors. This was investigated in this paper through the study of the spatial and temporal variations of benthic macroinvertebrates and water quality variables at the urban River Medlock in Greater Manchester, UK. Samples were obtained from five sections of the catchment (S1 to S5) over a period of 14 months and the results were compared with the standard requirement of the European Union’s Water Framework Directives. Multivariate tests including SIMPER (similarity percentages), PCA (principal component analysis) and BIOENV (biological and environmental) were carried out on the data in order to determine the environmental variables which most influenced the benthic macroinvertebrates. PCA of environmental variables indicated that 34% of the overall variance was heavily weighted on nutrients and catchment area (negatively on altitude and slope), 17% represented river substrate and the 12% represented discharge. The BIOENV analysis also indicated altitude, slope, catchment area, discharge and conductivity as the variables which influenced the biological communities. SIMPER analysis showed a difference between the upper and lower sections of the river with some sensitive taxa at the upper sites and showed that more organisms are present during spring. Apart from the lowest section of the river, the EU Water Framework Directive classification showed that other sites achieved the ‘good ecological status’. While 32 taxa groups were identified, abundant Baetidae, Chironomidae and Oligochaeta were recorded at all sites and seasons. The scores for biotic indices Whalley Hawkes Paisley and Trigg (WHPT) and Biological Monitoring Working Party (BMWP) were found to be similar. By the application of surrogate variables such as percentage urban cover, catchment area and total number of organism, the influence of urbanisation could be seen in the abundance of organisms over time and space.

## Introduction

Rivers provide services that are beneficial to all living organisms including cultural, provisional, regulatory and supporting roles. Although these ecosystem services are provided naturally, urban rivers are threatened by morphological adjustment, channel modifications and altered river landscapes, all of which affect stream health, ecological structure and function (Meyer et al. 2005; Paul and Meyer 2001; Walsh et al. 2005, 2012). Bank undercutting in urban rivers can reduce the habitat available for biological communities such as benthic macroinvertebrates in streams (Chadwick et al. 2010; Voelz et al. 2005). These modifications increase erosion rates, result in sediment production especially for lands that have been cleared for building and increase runoff into rivers leading to larger and more frequent floods. In urban rivers, changes to peak discharge, lag time, flood frequency and total runoff have been reported to increase the flashiness of the flow regime (Chin 2006).

While urban impacts on surface waters do not only occur at local and national scales, global impacts of urban rivers have been reported by Chin (2006) given the challenge of increasing human population and development. With increasing urban development, there is a pressing need to evaluate water quality in view of maintaining, protecting and restoring river organisms and biodiversity (Booth et al. 2005; Morley and Karr 2002).

As water is a resource which needs to be protected, maintained and restored, one of the most effective ways to determine stream health is to assess the benthic macroinvertebrates as biological indicators. This is due to their ease of collection for rapid assessments and their sensitivity to a range of stress including sewage pollution (e.g. Hellawell 1986; Lock et al. 2011; Metzeling et al. 2003). Among other biological variables, benthic macroinvertebrates are the most commonly used all over the world. In order to quantify biological status in rivers, biotic indices are applied as numerical expressions combining quantitative measures of species diversity with qualitative information on the ecological sensitivity of individual species (Czerniawska-Kusza 2004).

Biotic indices reveal what may not be shown by physicochemical variables (Purcell et al. 2002). For example, the index Biological Monitoring Working Party (BMWP) score system was developed for UK waters (Hawkes 1997) as a biological quality indicator to assess the pollution status of rivers. The BMWP index ranks individual macroinvertebrate families from 1 to 10 in increasing order of putative sensitivity to organic pollution, and the score is the sum of all scores from invertebrate families recorded in the sample (Hawkes 1997). The greater their tolerance to pollution, the lower the BMWP score system and vice versa (Armitage et al. 1983). Thus, the BMWP score is calculated based on the number of families sampled but not by abundances within those families. In order to address this gap, the European Union developed an index through the Water Framework Directive (‘WFD’, Directive 2000/60/EC) called the Whalley, Hawkes, Paisley and Trigg (Environment Agency 2015; WFD-UK Technical Advisory Group (UKTAG) 2014). The Whalley, Hawkes, Paisley and Trigg (WHPT) ASPT index (Environment Agency 2015) aims to integrate the abundance weighting limitation of the BMWP scoring system. The index identifies the effects of pressures on rivers with the ultimate aim to maintain or restore them to good ecological status as part of the WFD requirement. While the BMWP is based on the analysis of 82 taxa, the WHPT is based on 106 taxa so its sensitivity is slightly greater. The biological indicators of Biological Monitoring Working Party and the Whalley, Hawkes, Paisley and Trigg ASPT indices were applied to the results in order to assess the overall quality status while testing the differences in the outcomes. A river will achieve ‘Good Ecological Status’ classification for WFD when both physicochemical and biological variables are ‘good’. The lowest classification on any of the analysed parts is used to determine the overall status of the surface waters (‘WFD’, Directive 2000/60/EC).

Elosegi et al. (2017) suggest using three methods of ecosystem management (similar to those used in medicine)—diagnosis, treatment and prevention to effectively diagnose river conditions. In this study, the diagnosis was determined through spatial and temporal assessment for benthic macroinvertebrates and physicochemical variables in order to identify where adjustment process has taken place in the river that could have affected the density and diversity of the organisms. These variations within the catchment can imply different strategies and may be required to handle spatially distributed response mechanisms (Chin and Gregory 2005). The aims of this study are also to describe, measure and analyse the patterns of benthic macroinvertebrate communities’ assemblage in relation to study sites and environmental variables.

## Materials and methods

### Study site

**Fig. 1:**
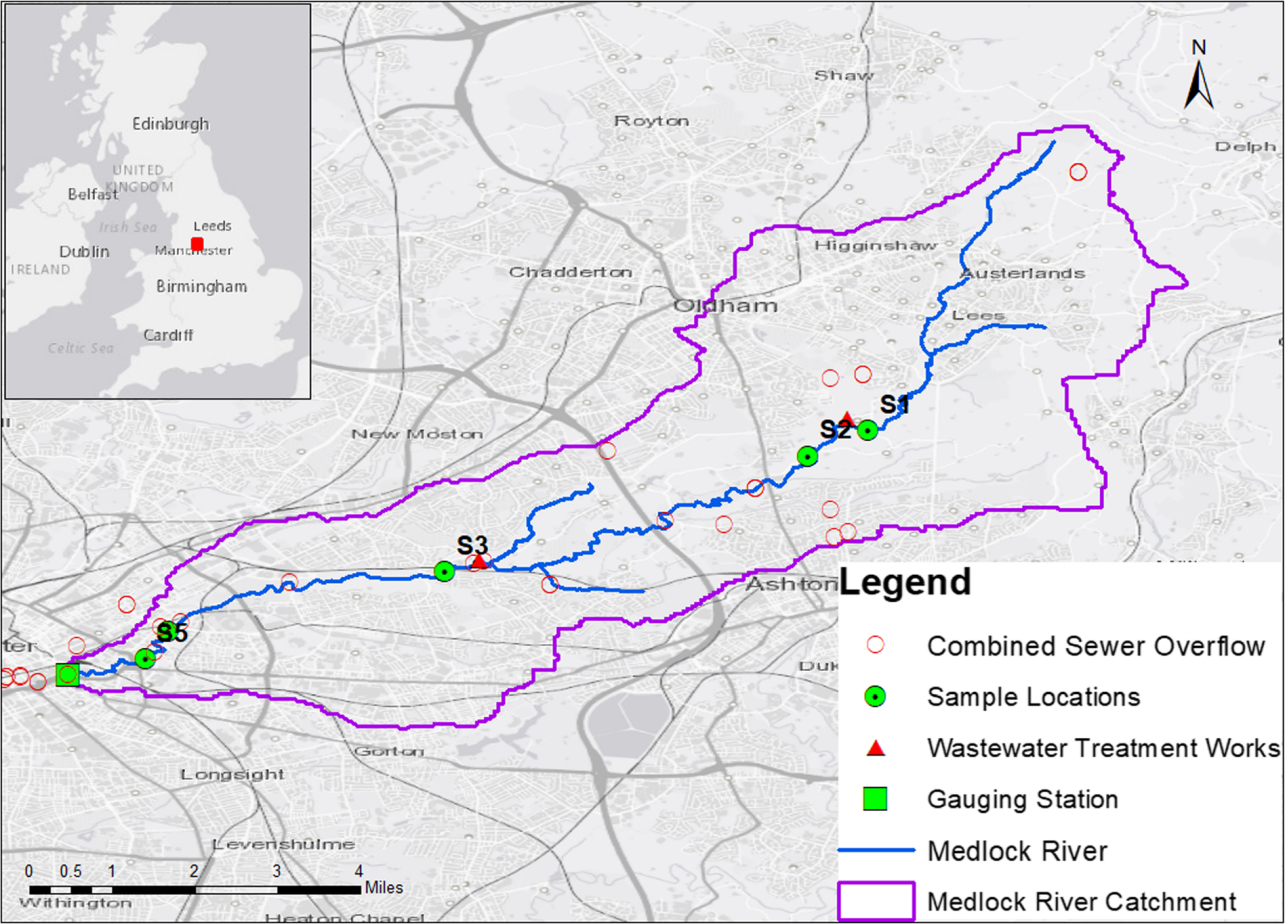
River Medlock catchment area and the study sites, S1 to S5. The sites are located upstream and downstream of the main wastewater treatment works and combined sewer overflows. The river’s gauge is located below site S5. Inset is the location of the river in Greater Manchester, UK

The River Medlock forms part of the 1776 square miles Mersey catchment and is one of the most urbanised in the UK (Environment Agency 2009). With a catchment area of 22.2 square miles and the average yearly flow rate is 0.82 m^3^ s^−1^ (CEH 2017), the River Medlock (see Fig. 1) is 13.67 miles in length and rises in the Pennine hills to the Northeast of Oldham in Greater Manchester (National Grid Reference (NGR): SD 95308 05431). It passes through a steep-sided wooded region for 6.21 miles before entering a largely urbanised area of Manchester city centre (NGR: SJ 85781 97858).

Much of the catchment is heavily urbanised (53%), but also consists of woodland and grassland comprising 43% of the catchment (CEH 2017). The surveyed reach of the river has a continuously operational wastewater treatment works (WwTW) at Failsworth (NGR: SJ 89674 99800), about 30 combined sewer overflows and numerous storm water overflows and surface water drains (EA, personal communication 2013).

Due to limited access to most parts of the river and given the small catchment area, a total of five sample sites (S1 to S5) were selected on the river upstream (sites S1 and S2) and downstream (sites S3 to S5) of the treatment works and combined sewer overflows (United Utilities, personal communication, 2013).

The catchment characteristics at each sub-section of the study sites including elevation, slope, dominant percentage land cover including grassland, built-up areas (comprising of urban and sub-urban cover) and woodlands are shown on Table 1. The Medlock catchment is dominated by grassland cover with the highest contribution at S1. S1 has an urban cover of 3.7% and the lowest catchment area when compared with other sample locations with higher urban covers. S1 also has the highest altitude and highest average slope. Appendix Table 10 shows equal distribution of the river substrate across sites with the dominant substrate recorded to be stones and sand.

**Table 1:** Catchment characteristics including the sub-catchment sampling sites S1–S5 and percentage contributions of the major land cover patterns at the study locations

Sub-catchment	Catchment characteristics				Grassland (%)	Woodland (%)	Built-up areas (%)	
	Average slope (%)	Sub-catchment area (miles^2^)	Minimum elevation (mMSL)	Altitude (m)			Sub-urban	Urban
S1	10.72	5.27	182	140	47.6	14.2	21.1	3.7
S2	9.45	7.93	159	107	40.8	14.1	20.7	10.5
S3	7.56	16.98	114	78.9	38.5	14.4	22.4	10.5
S4	7.24	19.37	93	36.1	36.8	14.6	22.8	12.3
S5	7.21	19.63	90	33.6	36.8	14.3	22.9	12.3

### Benthic macroinvertebrates

Monthly samples were obtained from the river between March 2013 and April 2014 for each of the five study locations and at four seasons including winter (December to February), spring (March to May), summer (June to August) and autumn (September to November). In order to obtain an accurate representation of total biodiversity (Armitage et al. 2012) across space and time, 3-min kick net sampling technique (Metzeling et al. 2003) was applied to collect benthic macroinvertebrates from the sites by using a 1-mm mesh net. An additional 1-min visual search was carried out to collect benthic invertebrates under stones that could have been missed through kick sampling (Murray-Bligh 2002). Replicates obtained at each site per sample period of 14 months were processed by using the qualitative standard method (Murray-Bligh 2002). Benthic macroinvertebrates were identified to family level in order to calculate the biotic indices including Biological Monitoring Working Party (BMWP) (Hawkes 1997) and Whalley, Hawkes, Paisley and Trigg (WHPT) (Clarke and Davy-Bowker 2014; Environment Agency 2015).

### Physicochemical variables

The variables—temperature, pH, dissolved oxygen (DO) and conductivity—were obtained at each site by using a pre-calibrated hand-held multiparameter water quality meter (YSi 556 Multi probe system YSI, Yellow Springs, OH, USA). Monthly spot samples were obtained at each site from March 2013 to April 2014 for the measurement of biochemical oxygen demand (BOD), phosphate-P (PO_4_-P), nitrate-N (NO_3_-N), ammonia-N (NH_3_-N) and suspended solid concentrations (mg/L).

For the measurement of nutrients, 300 ml of water sample was filtered through a 0.45-μm Millipore (Millipore-UK, Limited) hydrophilic, 47-mm cellulose acetate filter. The samples were processed within 24 h of collection and analysed using a *SEAL Auto Analyzer 3 High Resolution instrument* (SEAL Analytical Ltd., Southampton) based on a segmented flow analysis. For further information on the methods, see SEAL Analytical (2013). Throughout this study, concentrations of nutrients were presented as elemental concentrations, i.e. mg L^−1^ P, not PO_4_ and mg L^−1^ N, not of NO_3_; ammonia as mg L^−1^ of N not NH_3_. Detection limits for PO_4_-P measured as P was 0.004 mg L^−1^. Nitrate (NO_3_-N) measured as N following the DIN 38405 and ISO/DIS 13359 standard methods has a detection limit of 0.01 mg L**ˉ**^1^. NH_3_-N concentration (mg L^−1^) was analysed by spectrophotometry using the Hanna low range reagent kit HI-93700-01 (Hanna Instruments Ltd., Leighton Buzzard, Bedfordshire). The limit of detection for NH_3_-N measured as N was 0.01 mg L^−1^. All samples were processed by using the standard methods of analysis (Environment Agency 2011).

#### River discharge

The area ratio (AR) method (Archfield and Vogel 2010) was used to estimate river discharge at each sampling site using the following equation: *Y*_*ia*_ *= (A*_*y*_*/A*_*x*_*) X*_*ia*_, where *Y*_*ia*_ is the estimated discharge for month _*i*_ and year _*a*_ for site of interest; *A*_*y*_ is the catchment area of site of interest; *A*_*x*_ is the catchment area of gauging station; and *X*_*ia*_ is the discharge for month _*i*_ and year _*a*_ for site of interest for gauging station.

### Data analysis

The degree of similarity/differences between the benthic macroinvertebrate communities in relation to study locations and time was visualised using the similarity percentages (SIMPER) analysis. By applying the Bray Curtis dissimilarity metric, the species-abundance data were log (*x +* 1) transformed prior to SIMPER analysis and cutoff for low contributions was at 90.00%. All environmental variables including dissolved oxygen, temperature, conductivity, suspended solids, discharge, nutrients, river substrate, slope, altitude and sub-catchment areas were analysed using the principal component analysis (PCA) to characterize the physicochemical variation and benthic macroinvertebrates across the Medlock catchment. The first few PCs allow an accurate representation of the true relationship between the samples in the original high dimensional space as summarised by the percentage variation (Eigenvalues). The biological and environmental (BIOENV) analysis based on Spearman’s correlation matrix was used to determine which variable(s) affected benthic invertebrate abundance and distribution. All data matrix were square root transformed and normalised on a distance matrix to allow comparisons on the scale with the benthic macroinvertebrates and physicochemical variables. The weighed Spearman rank correlation coefficient (*ρ*) between the physicochemical variables and benthic invertebrate community similarity matrices formed the basis for this procedure. The physicochemical variable(s) with the largest *ρ* was taken to identify the best match with the benthic macroinvertebrates. All multivariate analyses were performed using PRIMER 6 (Clarke and Gorley 2006). Repeated measures ANOVA was determined for the environmental variables in GraphPad Prism version 8.2.1.

Benthic macroinvertebrates samples were analysed and interpreted by using the pollution indices-the Biological Monitoring Working Party (BMWP) scores, Average Score Per Taxon (ASPT) (Hawkes 1997) and Whalley Hawkes Paisley and Trigg (Clarke and Davy-Bowker 2014; Environment Agency 2015). Families investigated were BMWP scoring taxa present in more than 1% of samples. The WHPT ASPT was determined by dividing the sum of each taxon abundance by the WHPT number of taxa and each taxa has an abundance weighting which is unavailable for BMWP individual taxa.

## Results

### Benthic macroinvertebrates

#### Spatial analysis

The highest total number of organisms per site was recorded as follows: S1 (*n* = 877), S2 (*n* = 878), S3 (*n* = 914), S4 (*n* = 581) and S5 (*n* = 503) (Table 2). Sampling each of the five sites resulted in a total of 3387 benthic macroinvertebrates representing six classes, 11 orders and 32 families. The identified families were distributed across three phyla Annelida (worms and leeches), Mollusca (Gastropods and bivalves) and Arthropoda (insects and crustaceans). Arthropods contributed 23 invertebrate families, which made up 72% of the total composition. The density of organisms was found to be highest at site S1 which corresponds to the area of highest river altitude and lowest urban cover and sub-catchment area.

**Table 2:** Presence and absence of benthic macroinvertebrates along sites S1 to S5

Groups	S1	S2	S3	S4	S5
Hirudinea (Annelida)					
Erpobdellidae	3	3	6	11	2
Glossiphonidae			2	3	
Oligochaeta (Annelida)					
Lumbricidae	9	3	2	19	1
Lumbriculidae	69	2	167	14	22
Tubificidae	171	231	325	31	176
Crustacea (Arthropoda)					
Gammaridae	4	4	20	104	86
Asselidae	4	4	26	3	1
Trichoptera (Arthropoda)					
Rhyacophilidae	4	7	4	5	5
Hydropsychidae	5	8	7	10	7
Polycentropodidae	4			2	
Limnephilidae	1	26	3	14	
Psychomiyiidae		3		1	
Hydroptilidae		2			
Coleoptera (Arthropoda)					
Haliplidae				1	
Dytiscidae	3	3			
Hydrophilidae	1				
Ephemeroptera (Arthropoda)					
Baetidae	111	239	217	200	71
Ephemerellidae	18	19	3	1	
Heptageniidae	125	51	22	14	10
Leptophlebiidae	1		4		
Caeniidae		1			
Plecoptera (Arthropoda)					
Perlodidae	21	5			
Nemouridae	11				
Leuctridae	14	13			
Diptera (Arthropoda)					
Chironomidae	155	173	77	120	107
Simulidae	81	32	1	1	0
Tipulidae	40	29	21	21	14
Paediciidae	22	20		6	
Mollusca					
Sphaeridae			1		
Viviparidae			2		
Physidae			2		1
Lymnaeidae			2		
Total number of organisms	877	878	914	581	503
Density (organisms/miles^2^)	166	111	54	30	26

Among the insects, the Ephemeroptera taxa dominated the invertebrate assemblage with 85% contribution, Plecoptera contributed 5% and Trichoptera contributed 10% of organisms. Plecoptera was absent at the downstream stations S3 to S5 which could be linked to higher percentage cover, unsuitable substrate and contribution from contaminated urban runoff. Both sites S1 and S2 have lower % urban cover and had organisms that could be found in clean rivers including some organisms of the order Ephemeroptera, Plecoptera and Trichoptera. The metric to determine the ratio of Gammaridae to Asellidae was shown to be 1:1 at sites S1 and S2, but higher at S4 with 35:1 and at S5, it was 84:1.

Although the total number of organisms counted at S3 was higher than S2, S3 contributed more by the presence of Oligochaetes and molluscs. These groups of organisms are BMWP low scoring, pollution tolerant organisms found in poor quality waters, and could be found in areas with higher percentage of urban cover and finer river substrates. However, Oligochaeta was found at all sites making up 52% (comprising of 14% Lumbriculidae and Tubificidae, 38%) of the total taxa followed by Baetidae which accounted for 19% and Chironomidae contributed 17% of the total composition. A significant difference (*p* < 0.05) was shown to exist between all sample sites (ANOVA). In particular, the lower 95% confidence interval showed a difference between sites S1 and S5.

Between sites, SIMPER results showed that the average dissimilarity between the upper and lower sites, i.e. for S1 and S4 was recorded at 70% and between S1 and S5 was shown to be 69%. The differences between the upper and lower sites occurred due to higher contribution of Heptageniidae at S1 and Gammaridae at S4 and S5. Average dissimilarity between S1 and S3 was 67% also with Heptageniidae contributing at S1 but Tubificidae at S3. Apart from Heptageniidae which showed reduced numbers with increased catchment area and urban cover, other taxa groups including Baetidae, Tubificidae and Chironomidae showed increased abundance at the river (Table 2). The presence of Heptageniidae and Gammaridae organisms is associated with good and moderate river quality respectively while the presence of Tubificidae is indicative of diminished water quality. Within sites, the highest average similarity of organisms was found at site 4 (45%) with the highest contribution by the crustacean Gammaridae followed by Baetidae. All other sites had similar contribution with 40% similarity consisting of Baetidae and Chironomidae.

#### Percentage urban cover and abundance of taxa

**Fig. 2:**
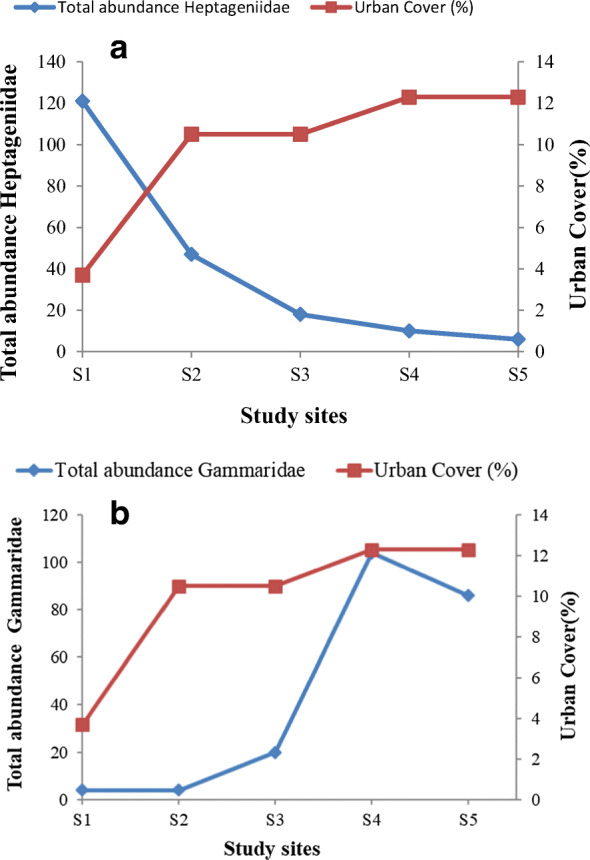
Percentage cover across study sites and **a** total abundance of Heptageniidae and **b** total abundance of Gammaridae

The relationship between selected taxa including Heptageniidae and Gammaridae and percentage urban cover is displayed on Fig. 2. While urban cover increased for each site downstream of the river at sites S3 to S5, there was a reduction in the total abundance of Heptageniidae, Gammaridae abundance increased with increasing urban cover suggesting the tolerance of the crustacean to diverse conditions.

PC1 for benthic macroinvertebrates (Appendix Table 11) accounted for 12% of the overall variance and was weighted on Limnephilidae, Ephemerellidae, Caeniidae, Perlodidae, Nemouridae, Leuctridae, Psychomyiidae and Hydroptilidae with values more than 0.2 while PC 2 accounted for 11% of the variance weighted on caddisflies Hydropsychidae. Each of these organisms represents high BMWP scoring mayflies (Ephemerellidae and Caeniidae) and other pollution sensitive organisms including caddisflies (Limnephilidae, Hydroptilidae, Psychomyiidae and Hydropsychidae) and stoneflies (Perlodidae, Nemouridae and Leuctridae). These benthic macroinvertebrates including cased caddisflies (Limnephilidae, Hydroptilidae, Psychomyiidae and Hydropsychidae), stoneflies (Perlodidae, Nemouridae and Leuctridae) and mayflies (Ephemerellidae and Caeniidae) had the highest record at sites S1 and S2.

The scores for BMWP and WHPT were determined for each site and the results showed that the scores obtained for both indices were similar (Table 3). The WHPT calculator accounts for each family in the taxonomic orders of Molluscs, Hemiptera, Odonata, Diptera, Trichoptera and Coleoptera. The presence and abundance per family in the listed groups imply that when more families are identified from among them, the higher the value allocated to them.

**Table 3:** Biotic indices—BMWP and WHPT scores and interpretation

			BMWP			WHPT		
Sites	BMWP NTAXA	BMWP score	Category	ASPT	Category	WHPT score	WHPT ASPT	WHPT NTAXA
S1	21	127	Good	6.05	Good	130.7	13.07	10
S2	19	114	Good	6	Good	124.3	10.34	12
S3	19	96	Good	5.05	Moderate	96.6	8.05	12
S4	17	86	Good	5.06	Moderate	93	8.45	11
S5	13	59	Moderate	4.53	Poor	49.7	9.94	5

For other taxonomic groups such as Ephemeroptera, Crustacea and Hirudinea, only a few families were included in the WHPT calculator. For example, the invertebrate families included in the WHPT calculator are Ephemerellidae and Caenidae but not for Heptageniidae and Baetidae, Oligochaetes and Plecoptera.

For BMWP, most families present in the taxonomic order were counted as number of taxa. The interpretation of BMWP scores based on Hawkes (1997) showed that all sites except site S5 had good biological quality. For ASPT classification, sites S1 and S2 were ‘good’, S3 and S4 were ‘moderate’ and S5 was ‘poor’. By applying the same interpretation to the WHPT indices, sites S1 to S4 would be classified as ‘good’ while site 5 would be considered ‘moderate’. For WHPT ASPT, all the sites would be classified as ‘very good’. This suggests that the WHPT index was more robust in interpretation better than BMWP. Statistically significant BMWP score showed degrading water quality from upstream, S1 to downstream sites S3 to S5 (ANOVA, *F*_4,62_ = 3.4, *p* < 0.05) and the difference was found between S1 and S5 (95% CI of difference = 4.2 to 38, *p* = 0.0071—Tukey’s test). Average Score Per Taxon (BMWP ASPT) also showed a statistically significant (ANOVA, *F*_4,62_ = 3.2, *p* < 0.05) difference between the sites and in particular between S1 and S3 (95% CI of difference = 0.072 to 3, *p* = 0.0350—Tukey’s test). Between sites, WHPT ASPT showed significant differences (ANOVA, *F*_4,62_ = 5.6, *p* < 0.05) between sites and Tukey’s multiple comparison test shows the difference between S1 and S3 (95% CI of difference = 0.47 to 2.7, *p* = 0.0016); S1 and S4 (95% CI of difference = 0.032 to 2.2, *p* = 0.0407); S1 and S5 (95% CI of difference = 0.2 to 2.5, *p* = 0.0127) and between S2 and S3 (95% CI of difference = 0.079 to 2.3, *p* = 0.029).

#### Temporal analysis

**Fig. 3:**
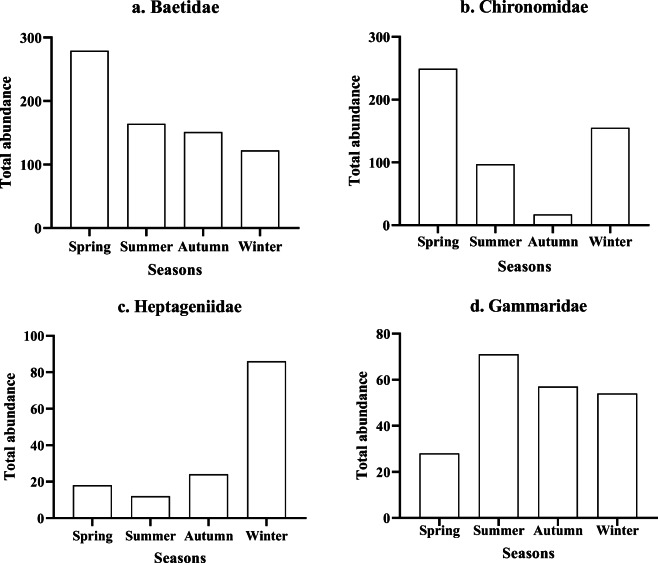
Total abundance of organisms with 10% contribution between seasons. **a** Baetidae had the highest contribution during spring but declined gradually towards winter. **b** Chironomidae had a corresponding high contribution during spring, but had the lowest record at autumn. **c** Heptageniidae had similar contribution during spring and winter while **d** Gammaridae had the highest contribution during the summer and the lowest at spring

The total number of organisms obtained during the seasons are recorded in decreasing order: spring (1413) > winter (1082) > summer (519) > autumn (355). The highest and lowest records of organisms were found during spring and autumn respectively (see Appendix Table 12). For specific organisms, Fig. 3 shows organisms with 10% contribution with the highest contributions between seasons including Baetidae, Chironomidae, Gammaridae and Heptageniidae. The highest average dissimilarity (65%) of organisms was found between summer and autumn (Appendix Table 13) and this was distinct due to the presence/abundance of Chironomidae and Heptageniidae.

Within each season, Table 4 shows average similarity for benthic macroinvertebrates to be highest for spring (49%) followed by autumn (37%) and the lowest average similarity occurred during winter (34%). For specific organisms, Baetidae had the highest percentage similarity contribution during autumn at 66% > summer 42% > spring 35% > winter 20%. The next contributing taxa was Chironomidae which had the highest record during spring 26% > summer 19.9% > winter 16% > autumn 8.4%. The ratio of Gammaridae to Asellidae was shown to be 3.3:1 during spring and winter, 18:1 during summer and 11:1 at autumn. These results imply that Gammaridae was abundant in the river compared to Asellidae at higher temperatures which favoured their growth.

**Table 4:** Average abundance and similarity of organisms within each season from March 2013 to April 2014 for spring (March–May), summer (June–August), autumn (September to November) and winter (December to February). Figures in italics highlight organisms with more than 10% contribution to the total organisms present at each season

Spring average similarity, 49.17	Contribution (%)	Winter average similarity, 33.50	Contribution (%)
*Baetidae (E)*	*34.8*	*Baetidae (E)*	*19.53*
*Chironomidae*	*25.5*	*Chironomidae*	*15.96*
*Heptageniidae (E)*	*10.5*	*Tipulidae*	*15.41*
*Gammaridae*	*10.2*	*Heptageniidae (E)*	*11.63*
Tubificidae	3.93	Hydropsychidae (T)	7.63
Tipulidae	3.35	Tubificidae	6.69
Lumbriculidae	3.18	Simulidae	4.85
Autumn average similarity, 37.14	Contribution (%)	Lumbriculidae	3.9
*Baetidae (E)*	*66.1*	Erpobdellidae	3.66
Chironomidae	8.31	Gammaridae	3.42
Gammaridae	7.91		
Heptageniidae (E)	6.68		
Hydropsychidae (T)	3.37		
Summer average similarity, 36.50	Contribution (%)		
*Baetidae (E)*	*42.2*		
*Chironomidae*	*19.9*		
*Gammaridae*	*10.5*		
Ephemerellidae (E)	7.3		
Tubificidae	3.49		
Heptageniidae (E)	3.38		
Lumbriculidae	2.23		
Rhyacophilidae (T)	1.91		

Ephemeroptera (E); Plecoptera (P) and Trichoptera (T)

BMWP scores (Table 5) showed that the river’s status based on BMWP scores was good at all seasons while the ASPT showed the river’s condition to be ‘moderate’. When the same classification was applied to WHPT calculator, the river also achieved the status ‘very good’ biological quality as ASPT values were more than 8. Between seasons, BMWP scores and ASPT showed no significant (*p* > 0.05) difference.

**Table 5:** Biotic indices—BMWP and WHPT scores, number of taxa (NTAXA) and average score per taxon (ASPT) determined for the sites on the basis of abundance

	BMWP					WHPT		
Season	BMWP score	Score category	BMWP ASPT	ASPT category	BMWP NTAXA	WHPT score	WHPT NTAXA	WHPT ASPT
Spring	115	Good	5.23	Moderate	22	127	14	9.1
Summer	118	Good	5.9	Moderate	20	124	12	10.33
Autumn	103	Good	5.4	Moderate	19	108.5	12	9.042
Winter	86	Good	5.37	Moderate	16	86.4	10	8.64

### Environmental variables

#### Spatial analysis and PCA of environmental variables

The mean and standard deviation for the variables measured at the study river (see Table 6) indicated that all river sections sampled had high percentage oxygen saturation, normal range pH and temperature conditions. Conductivity levels ranged from 484 to 693 μS cm^−1^ with the lowest value at site S1 and the highest at S5. The average concentration of BOD was less than 5 mg L^−1^, NH_3_-N < 0.6 mg L^−1^ and PO_4_-P concentration which was more than 0.12 mg/L at sites S3 to S5. Classification of the river on the basis of WFD designation showed the river to be of ‘poor’ quality due to the concentration (mg/L) of PO_4_-P.

**Table 6:** Mean ± standard deviation of physicochemical variables at sites S1 to S5

	S1	S2	S3	S4	S5
DO (% saturation)	104.5 ± 7.90	100 ± 8.62	102.8 ± 10.34	100.8 ± 9.85	100.1 ± 12.03
pH (pH units)	8.0 ± 0.44	7.8 ± 0.29	8.1 ± 0.22	8.1 ± 0.26	8.1 ± 0.20
Temperature (°C)	9.7 ± 3.32	9.6 ± 3.26	10.7 ± 4.06	10.3 ± 4.13	10.3 ± 4.29
Conductivity (μS cm^−1^)	484 ± 129.92	559.7 ± 143.95	650.3 ± 149.80	684.5 ± 153.72	693.7 ± 154.61
Suspended solids (mg L^−1^)	4.2 ± 6.20	6.0 ± 5.77	11.6 ± 14.70	15.0 ± 26.68	12.1 ± 19.78
Discharge (m^3^ s^−1^)	0.15 ± 0.12	0.23 ± 0.18	0.43 ± 0.34	0.53 ± 0.41	0.53 ± 0.41
BOD_5_ (mg L^−1^)	2.0 ± 2.83	2.3 ± 2.62	2.9 ± 3.02	3.3 ± 3.20	2.3 ± 1.32
NH_3_-N (mg L^−1^)	0.4 ± 0.55	0.5 ± 0.52	0.6 ± 0.58	0.5 ± 0.51	0.5 ± 0.58
NO_3_-N (mg L^−1^)	0.9 ± 1.09	1.1 ± 1.17	4.0 ± 3.04	4.3 ± 2.54	4.1 ± 2.27
PO_4_-P (mg L^−1^)	0.1 ± 16	0.1 ± 0.25	0.6 ± 0.42	0.5 ± 0.34	0.5 ± 0.31

Table 7 shows that PC1 accounted for 34% of the overall variance and was most heavily weighted on PO_4_-P, NO_3_-N and catchment area. PC 2 accounted for 18% of the variance representing variations in river substrate specifically boulders and stones while PC3 was dominated by conductivity and discharge with a variance of 12%. Discharge would influence the transport of substrate and nutrients faster across the river length to areas with reduced slope and altitude. With increased catchment area, water supplies could be abstracted for public use, urban runoff and effluent from housing and industrial development could increase contamination in wider catchment areas and more urbanised sites. These artificial influences operating within a larger catchment could alter natural runoff and therefore impact on flow and dilution of variables as demonstrated by sites S3 to S5.

**Table 7:** Results of principal component analysis obtained for physicochemical and hydromorphological variables at the sampling locations

Variable	PC1 (34%)	PC2 (18%)	PC3 (12%)
DO (% saturation)	− 0.072	− 0.06	0.231
pH (pH units)	0.141	− 0.081	− 0.295
Temperature (°C)	0.022	− 0.001	− 0.444
Conductivity (μS cm^−1^)	0.115	0.02	*0.333*
BOD	0.138	0.025	− 0.024
NH_3_-N (mg L^−1^)	0.045	0.081	0.42
NO_3_-N (mg L^−1^)	*0.304*	0.008	− 0.259
PO_4_-P (mg L^−1^)	*0.318*	− 0.007	− 0.208
Suspended solids (mg L^−1^)	0.148	0.18	0.185
Discharge (m^3^ s^−1^)	0.212	0.116	*0.451*
Catchment area (miles^2^)	*0.372*	0.071	− 0.001
Boulders (%)	− 0.007	*0.485*	− 0.074
Stones (%)	− 0.168	*0.474*	− 0.071
Pebbles (%)	− 0.356	0.124	− 0.055
Gravel (%)	0.136	− 0.098	0.077
Sand (%)	0.15	*− 0.445*	0.05
Silt (%)	− 0.144	*− 0.487*	0.059
Altitude (m)	*− 0.368*	− 0.051	0.008
Slope (%)	*− 0.364*	− 0.061	0.002

#### Temporal analysis of environmental variables

Significant differences were found for pH *F*(1.436, 5.746) = 18.11, *p* = 0.0043; temperature *F*(1.033, 4.131) = 220, *p* < 0.0001; conductivity *F*(1.059, 4.235) = 35.76, *p* = 0.0032; BOD, *F*(1.338, 5.354) = 12.34, *p* = 0.0127; NH_3_-N *F*(2.026, 8.103) = 47.76, *p* < 0.0001 and TOM, *F*(1.268, 5.073) = 17.08, *p* = 0.0075. During these seasons, the highest levels of pH and BOD were recorded during summer, NH_3_-N and TOM levels were highest during winter and conductivity level was highest during autumn. There were no significant (*p* > 0.05) differences found for NO_3_-N, PO_4_-P and suspended solids.

### Benthic macroinvertebrate assemblages and environmental variables

Biological and environmental (BIOENV) test performed for environmental variables and benthic macroinvertebrate communities (Table 8) showed a weak correlation *ρ* = 0.274 for five out of 20 variables. These five variables including conductivity, river discharge, catchment area, altitude and slope were shown to be the most important variables controlling the benthic macroinvertebrate abundance/richness. Although the correlation value was low (0.274), the iterations suggest that nutrient concentration (mg/l) especially PO_4_-P had no major influence on the abundance of benthic invertebrate community in this river. Instead, the relationship between the identified variables showed an increase in conductivity as discharge increased suggesting the impact of point source contribution; a strong correlation between discharge and catchment area suggests the impact of urban off and other point source contributions. However, as the slope decreased, the river discharge increased. Also, at high altitude, the percentage urban cover was lowest and the river concentrations were lowest which suggests an inverse relationship between altitude and catchment area.

**Table 8:** Weighted Spearman’s rank correlation using between biotic and abiotic variables using the BIOENV procedure

Number of variables	Weighted Spearman’s rank (*ρ*)	Variables
5	0.274	Conductivity, discharge, catchment area, altitude, slope
4	0.273	Conductivity, discharge, catchment area, altitude
5	0.272	Conductivity, phosphate-P, discharge, catchment area, altitude
2	0.272	Conductivity, catchment area
5	0.271	Conductivity, nitrate-N, discharge, catchment area, altitude

### Chronosequence of physicochemical variables and abundance of benthic macroinvertebrate assemblages

**Fig. 4:**
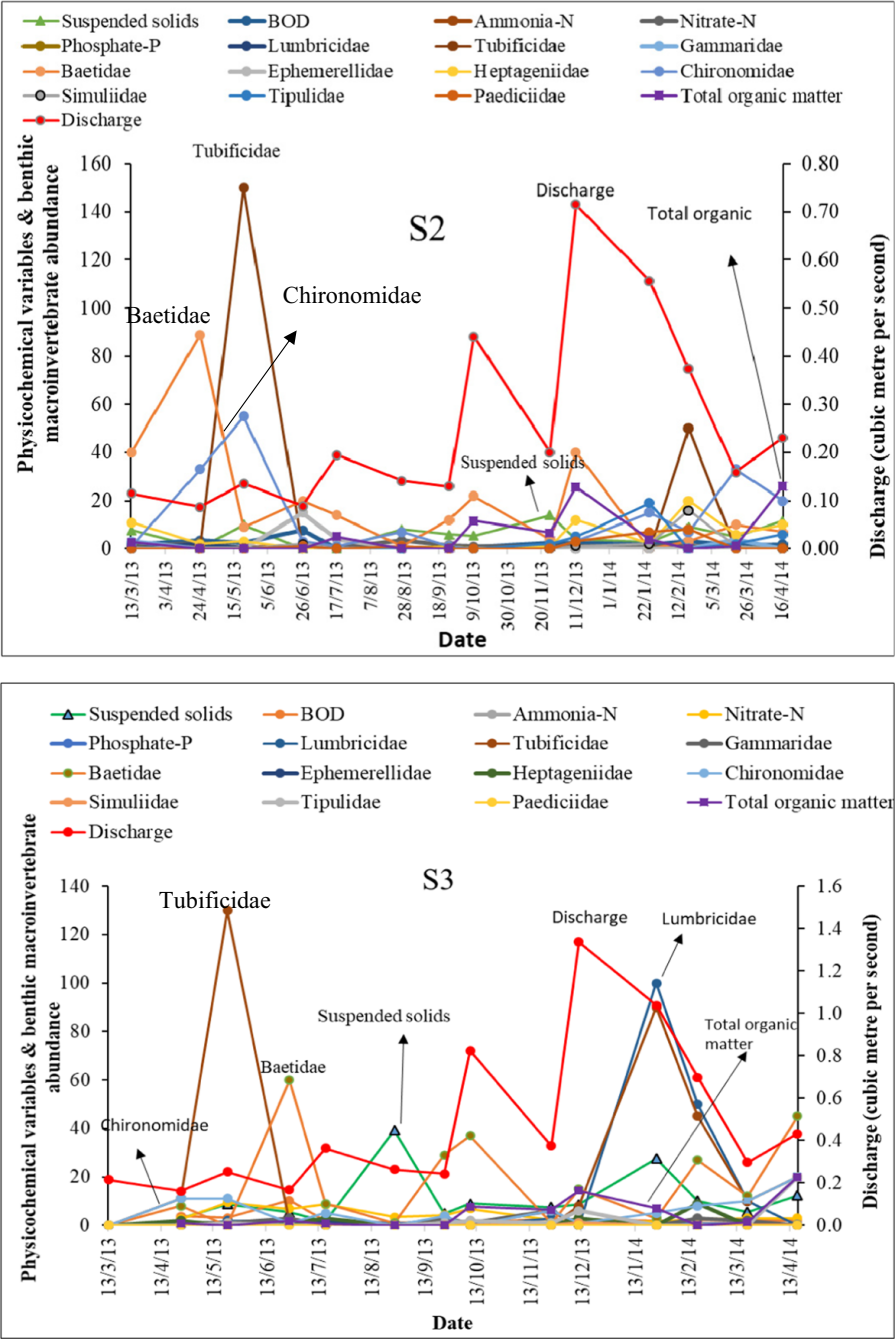
Benthic macroinvertebrate abundance and physicochemical variables from a chronosequence of sites S2 and S3 which are upstream and downstream of a major WwTW. Data from the sample period was a full year from 13 March 2013 to 13 April 2014. Variables with the highest count were re-identified on the graphs

A time series of water chemistry, river discharge and benthic macroinvertebrates was analysed for sites S2 and S3 respectively (Fig. 4). The sites S2 and S3 are located upstream and downstream of the wastewater treatment works.

At site S2, the river discharge increased from 0.11 m^3^/s in 13 March 2019 (spring) until a peak of 0.72 m^3^/s in 11 December 2013 (winter) and gradually declined to 0.23 m^3^/s in 13 April 2014 (spring).

The highest concentration of suspended solids (14 mg/l) was recorded in 13 November 2019 (autumn) and for ammonia-N (0.9 mg/l) was recorded in 13 February 2014 (winter). All other levels of physicochemical variables recorded at S2 were low. The benthic macroinvertebrate which dominated the river was the mayfly, Baetidae, as the total number counts were distributed across the sample months. The highest count of Baetidae was recorded in spring (13 April 2013). This was followed by the worm, Tuficidae and in particular, 13 May 2013 (spring) showed an abundance of 150 total counts of Tubificidae at a discharge of 0.14 m^3^/s. With increased discharge, the assemblage of Tubificidae reduced until 13 February 2014 (winter) where 50 counts were recorded at a discharge level of 0.37 m^3^/s. Followed by Tubificidae is the taxa Chironomidae which had 89 and 55 counts in April and May respectively. Other taxa including Ephemerellidae were recorded in June (13 June 2013) and Heptageniidae, Paediciidae and Tipulidae were recorded from late winter to spring (February to April) of 2014 when the concentration of total organic matter was highest.

At S3, high concentration of suspended solid concentration (39 mg/l) was recorded in 13 August 2013 (summer) followed by a concentration of 28 mg/l which was 13 January 2014 (winter) the following year. The river discharge increased from 0.21 m^3^/s to a peak of 1.34 m^3^/s on 11 December 2013 (winter). The taxa Tubidicidae was abundant at S3 with peaks in 13 May 2013 (spring) and 13 January 2014 (winter). This was followed by Baetidae. The highest count for the worm Lumbricidae was recorded in 12 January 2014 (winter).

Nutrient concentration at S3 was higher than the records at site S2. The average phosphate-P concentration recorded at S3 was 0.58 mg/l compared to 0.08 mg/l recorded at S2; average nitrate-N concentration recorded at S3 was 4.33 mg/l and at S2, this was 1.03 mg/l; average ammonia-N concentration recorded at S3 was 2.76 mg/l and at S2, this was 2.07 mg/l; average concentration of suspended solids recorded at S3 was 11.03 mg/l and at S2, this was 5.98 mg/l. Average river discharge recorded at S3 was 0.48 m^3^/s and at S2, this was 0.25 m^3^/s.

The results from the two sites showed that Baetidae, Tubificidae and Chironomidae were abundant during spring when the river discharge was low but could be found in less abundance during winter when the river discharge was highest. The concentration of suspended solids was highest during summer when the river discharge was lowest. Highest average concentration of total organic matter (11.96 mg/l) was recorded at S2 between October (autumn) and January (winter) while S3 had a mean concentration in the same period of 8.83 mg/l). Therefore, S2 will support more functional feeding groups such as shredders and grazers during autumn and winter seasons. These results correspond the findings of the ‘Temporal analysis’ and ‘Temporal analysis of environmental variables’ sections.

## Discussion

### Spatial variables

Both benthic macroinvertebrates and physicochemical variables showed differences between the sites upstream and downstream of the wastewater treatment works. Abundant benthic macroinvertebrates and more environmentally sensitive organisms were found at the upper sites S1 and S2. These sites had the lowest concentration of physicochemical variables and high dissolved oxygen levels. On the basis of WFD classification, these sites would be considered to have achieved the ‘good ecological status’. However, a river catchment is classified as a whole and on the basis of the weakest contributing variable (European 2000). Thus, a higher concentration of PO_4_-P concentration recorded at the lower sites S3 to S5 led to a ‘poor’ chemical status. High PO_4_-P concentration has been reported as a major challenge in this catchment (James et al. 2012) and the main pollution sources to the more urbanised sections of the river include the effluent received from the major treatment works, combined sewer overflows and diffuse pollution from leaked pipes and contaminated urban runoff. Similar patterns of downstream impacts of PO_4_-P concentration from treatment works have been found in other urban rivers as reported by Bowes et al. (2008), Jarvie et al. (2008); Morley and Karr (2002), Neal et al. (2005), Paul and Meyer (2001) and Walsh et al. (2001). Higher sub-catchment areas and urban covers were recorded at the locations downstream (sites S3 to S5) of the treatment works and these results suggest these locations will be impacted by other factors such as silted banks and modified river channels. These factors will impact on the river’s ability to effectively perform its ‘ecosystem service’ including the maintenance of biodiversity (Brown 2007). Following this development, the river will not achieve ‘good ecological status’ as required by the EU WFD.

S1 and S2 had higher biotic scores and are classified as having ‘good’ quality status. Given the condition of S1 which was classified as ‘good’ for both physicochemical and ecological variables, this site could be used as a reference site to compare with other sections of the river. However, this may not be ideal because this section also receives effluent from CSOs and leachates from agricultural sites and therefore reveals the challenge of obtaining a control river site for studies in urban areas. The selection and application of a reference site in order to assess urban influence on parts of two Colorado river invertebrates have been studied (Voelz et al. 2005). Previous study of benthic invertebrates at River Medlock by Frost et al. (1976) showed that pollution tolerant Baetidae, Chironomidae and Oligochaeta had always been abundant especially at the lower reaches. Hence, after four decades and the operation of light industrial pollution from the city and post WFD implementation, these taxonomic groups still dominate the system, but the presence of moderately tolerant Gammaridae at the lower reaches indicates some improvement.

### Temporal variation

The total taxon richness was higher in spring and winter than it was for summer and autumn. The percentage contribution for EPT was highest during spring with 94% contribution from Ephemeroptera (with dominance by Baetidae), 0.9% Plecoptera and 4.8% for Trichoptera. The river’s state of health was discriminated for study sites by the BMWP scores, higher discharge at the lower sites and the transport of nutrients and conductivity downstream of the river thereby suggesting the impact of other factors. The highest score for biotic indices was recorded during spring while the lowest score was recorded during winter. The reasons for the seasonal differences were attributed to the growing spring season which suggests abundant food for benthic macroinvertebrates. Although the highest dissimilarity of organisms was found between summer and winter (69%), summer low flow leading to limited dilution, high nutrient influx could enhance abundant environmentally tolerant taxa and during winter increased flow will lead to increase urban runoff, high conductivity due to increased salt application to main roads and pathways. These conditions will favour organisms such Baetidae, Chironomidae and Oligochaeta which were in higher numbers. Other studies have found these groups to dominate the macroinvertebrate communities (Brittain et al. 2001; Guimaràes et al. 2009) especially in urban river systems (Minshall 1988; Townsend et al. 1997). During autumn, the highest level of conductivity recorded corresponds to the lowest total number of organisms recorded for the same period. This further suggests that high levels of ions do not favour the abundance of benthic macroinvertebrates.

The ratio of Gammaridae to Asselidae was found to be highest during summer when the temperature was warm. Hynes (1955) showed that under summer conditions (10–15 °C), Gammaridae could mature between 3 and 4 months and during winter conditions (5–10 °C), this would be about 7 months. During spring and winter, therefore, Gammaridae would be subject to heavy mortality or would be their resting stages.

### Benthic macroinvertebrates and physicochemical variables

The relationship between benthic macroinvertebrates and physicochemical variables indicates that geomorphological variables co-vary with river discharge, river substrate and conductivity along the river sites which could influence their fauna assemblage. PCA for benthic macroinvertebrates (Appendix Table 11) showed that environmentally sensitive families structured the principal components. Therefore, the presence of these organisms suggests that the river was not influenced by environmental pollution. Echols et al. (2009) found mayflies were dominant where conductivity levels were lowest and show that high levels of conductivity could influence invertebrate numbers in a river. Some studies including Kefford (1998) and Roy et al. (2003) found a relationship between conductivity and benthic macroinvertebrates suggesting that a consistently elevated total dissolved ions may lead to biotic impairment of surface waters. Other sources of contamination including urban runoff, overflows and effluent from point sources, e.g. the wastewater treatment works, contribute to high concentrations of salts in the more urbanised sections of the river. Similar patterns have been found in urban rivers as reported by Morley and Karr (2002), Paul and Meyer (2001) and Walsh et al. (2001).

Chronosequence analysis (‘Chronosequence of physicochemical variables and abundance of benthic 415 macroinvertebrate assemblages’ section) supported the fact that the river was mostly influenced by the discharge contributions received from the WwTW above S3 and inflows from urban runoff at S3. Furthermore, increasing levels of discharge and nutrient concentration at S3 also supported the overflow contribution from combined sewers. These conditions influenced the abundance of environmentally tolerant taxa groups such as Oligochaeta, Baetidae and Chironomidae with less diversity of sensitive taxonomic groups, e.g. Heptageniidae and Ephemerellidae. While these results corroborate the findings of the ‘Temporal analysis’ section, a higher concentration of total organic matter recorded at site S2. These showed that S2 would favour functional feeders such as scrapers (e.g. Heptageniidae), shredders (e.g. Ephemerrelidae, Tipulidae) which are abundant in places with higher organic matter in particular leaf litter (Ramírez and Gutiérrez-Fonseca 2014) and indicates that S2 is more rural when compared with urban S3.

Altitude and slope correlated negatively on the PC 1 analysis and were found to influence the distribution of benthic macroinvertebrates. Environmentally sensitive EPT organisms were found at the locations of high altitude and slope in particular at sites S1 and S2. Although altitude and slope may co-vary in this study catchment, their differences may not be quite separable given the small catchment size. However, they may be influenced other variables such as increased discharge regimes which influence nutrient release through allochthonous energy influx (Bispo and Oliveira 2007; Vanessa et al. 2014) through the movement of substrate downstream of the river. These factors modify the composition and abundance of benthic macroinvertebrates (Miserendino 2001; Skoulikidis et al. 2009).

### Overall classification

Both BMWP and WHPT indices showed the river to be ‘very good’ based on the WFD classification. With similarities in scores for BMWP and WHPT, both indices could provide a robust explanation by identifying selected organisms which could increase the river’s taxa and therefore explain the state of the river at any given time. While BMWP index is simpler to apply in assessing pollution status (Roche et al. 2010) and the method of interpretation was found to be easier for the non-technical specialist, the WHPT index considers the abundance per taxa of certain groups of organisms in order to work out the scores.

Physicochemical variables such as high dissolved oxygen saturation, moderate concentration of BOD and NH_3_-N could increase the abundance of sensitive benthic macroinvertebrates and by proxy, the biotic indices across sites. The results obtained from this study show that sewage pollution is not a major challenge in this river apart from the higher concentration (mg/L) of PO_4_-P. This result is in line with the report produced by the UK Environment Agency on the catchment classification (Table 9) under the WFD compliance scheme. It showed the river to be ‘poor’ in 2014 but has progressed to ‘moderate’ status in 2016 and all variables are predicted to be ‘good’ by 2027. The reasons for not achieving good status were attributed to intermittent sewage discharge (for benthic macroinvertebrates) and from urban development which caused the high phosphate concentration. (See https://environment.data.gov.uk/catchment-planning/WaterBody/GB112069061151.) In order to achieve ‘good ecological status’, the environmental regulators will consider a need to balance the management priorities in relation to the cost of management and the extent of burdens on the river.

**Table 9:** WFD classifications from 2015 to 2021 cycle for the River Medlock

Classification item	2013	2014	2015	2016		2027
Overall water body	Moderate	Poor	Moderate	Moderate		Good
Ecological	Moderate	Poor	Moderate	Moderate		Good
Chemical	Good	Good	Good	Good		Good

**Table 10:** Mean river sediment substrate at sites S1 to S5

Substrate	Boulders (%)	Pebbles (%)	Stones (%)	Gravel (%)	Sand (%)	Silt (%)
S1	5.50	6.50	36.50	4.50	30.00	15.00
S2	10.00	4.50	39.09	6.50	28.64	12.27
S3	4.00	1.00	27.00	22.50	32.00	12.22
S4	13.50	2.50	43.00	6.00	27.50	7.50
S5	5.63	1.43	17.50	3.75	41.88	15.63

**Table 11:** PCA values obtained for benthic macroinvertebrate assemblages at the sampling locations

Variable	PC1 (11.9%)	PC2 (11.1%)	PC3 (8.7%)
Erpobdellidae	− 0.23	0	− 0.131
Glossiphonidae	− 0.161	0.133	− 0.047
Lumbricidae	− 0.171	0.169	− 0.149
Lumbriculidae	− 0.108	− 0.049	− 0.022
Tubificidae	− 0.153	− 0.298	− 0.159
Gammaridae	− 0.164	0.122	0.136
Asselidae	− 0.185	0.136	− 0.076
Rhyacophilidae	0.036	0.134	− 0.236
Hydropsychidae	− 0.047	*0.218*	− 0.188
Polycentropodidae	− 0.106	0.185	− 0.202
Limnephilidae	*0.212*	0.063	− 0.161
Haliplidae	− 0.018	0.087	0
Dytiscidae	0.145	− 0.197	− 0.144
Hydrophilidae	− 0.017	− 0.197	− 0.125
Baetidae	0.034	0.165	− 0.009
Ephemerellidae	*0.354*	− 0.027	− 0.195
Heptageniidae	− 0.058	0.177	− 0.31
Leptophlebiidae	− 0.136	− 0.443	− 0.152
Caeniidae	*0.229*	− 0.019	0.154
Perlodidae	*0.302*	− 0.112	− 0.251
Nemouridae	*0.21*	− 0.095	− 0.172
Leuctridae	*0.381*	− 0.019	− 0.043
Psychomyiidae	*0.32*	0.005	−0.06
Hydroptilidae	*0.229*	− 0.019	0.154
Chironomidae	− 0.077	− 0.114	− 0.122
Simulidae	− 0.005	0.119	− 0.327
Tipulidae	−0.155	0.139	− 0.307
Paediciidae	0.073	0.064	− 0.405
Sphaeridae	− 0.143	− 0.394	− 0.106
Viviparidae	−0.143	− 0.394	− 0.106
Physidae	− 0.043	− 0.02	0.083
Lymnaeidae	0.03	− 0.004	0.048

**Table 12:** The presence and absence of benthic macroinvertebrates across the four seasons: spring (March–May), summer (June to August), autumn (September to November) and winter (December to February)

	Spring	Summer	Autumn	Winter
Hirudinea (Annelida)				
Erpobdellidae	7		6	12
Glossiphonidae	1	1	1	2
Oligochaeta (Annelida)				
Lumbricidae	17	7		10
Lumbriculidae	28	9	38	199
Tubificidae	565	7	3	209
Crustacea (Arthropoda)				
Gammaridae	36	71	57	54
Aselidae	11	4	5	18
Trichoptera (Arthropoda)				
Rhyacophilidae	11	4	1	9
Hydropsychidae	5	3	13	16
Polycentropodidae	4	1		1
Limnephilidae	1	42		1
Psychomiidae		2	1	1
Hydroptilidae			2	
Coleoptera (Arthropoda)				
Haliplidae	1			
Dytiscidae	2	3	1	
Hydrophilidae	1			
Ephemeroptera (Arthropoda)				
Baetidae	326	164	151	122
Ephemerellidae	0	39	1	1
Heptageniidae	80	12	24	86
Leptophlebiidae	5			
Caeniidae			1	
Plecoptera (Arthropoda)				
Perlodidae	4	22		
Nemouridae		11		
Leuctridae		10	17	
Diptera (Arthropoda)				
Chironomidae	292	97	17	155
Simulidae	9	3	1	82
Tipulidae	16	1	11	67
Paediciidae	5	4	2	37
Mollusca				
Sphaeridae	1			
Viviparidae	2			
Physidae	1		2	
Lymnaeidae		2		
Total number of organisms	1431	519	355	1082

**Table 13:** Average dissimilarities (SIMPER) between seasons spring (March to May), summer (June–August), autumn (September to November) and winter (December to January) based on average abundance and percentage contribution of benthic macroinvertebrate during study period. Figures in italics indicate organisms with 10% contribution to the total counts

Summer and autumn average dissimilarity = 64.77	Contribution (%)	Spring and winter average dissimilarity = 60.75	Contribution (%)
*Chironomidae*	*10.58*	*Baetidae*	*9.67*
*Gammaridae*	*9.72*	Chironomidae	9.3
*Heptageniidae*	*7.28*	Gammaridae	8.54
Ephemerellidae	6.76	Heptageniidae	8.49
Baetidae	6.71	Tubificidae	8.4
Tubificidae	6.6	Tipulidae	7.98
Lumbriculidae	5.44	Lumbriculidae	7.4
Leuctridae	4.65	Simulidae	6.07
Hydropsychidae	4.39	Hydropsychidae	5.41
Asselidae	4.05	Asselidae	5.03
Lumbricidae	3.74	Paediciidae	4.63
Tipulidae	3.65	Lumbricidae	4.48
Erpobdellidae	3.34	Erpobdellidae	4.45
Limnephilidae	3.02	Rhyacophilidae	3.52
Paediciidae	3		
Rhyacophilidae	2.85		
Dytiscidae	2.4		
Perlodidae	2.08		
Spring and summer average dissimilarity = 59.57	Contribution (%)	Spring and autumn average dissimilarity = 58.59	Contribution (%)
Chironomidae	8.98	*Chironomidae*	*13.11*
Heptageniidae	8.61	*Heptageniidae*	*10.38*
Tubificidae	8.6	*Gammaridae*	*9.79*
Gammaridae	8.4	Tubificidae	7.74
Lumbriculidae	5.99	Lumbriculidae	7.34
Baetidae	5.87	Tipulidae	6.79
Ephemerellidae	5.79	Baetidae	6.36
Tipulidae	5.34	Asselidae	6.23
Lumbricidae	5.08	Hydropsychidae	4.56
Asselidae	4.68	Lumbricidae	4.3
Rhyacophilidae	3.43	Erpobdellidae	4.24
Simulidae	3.41	Paediciidae	4.03
Erpobdellidae	3.29	Simulidae	2.85
Paediciidae	3.07	Rhyacophilidae	2.18
Leuctridae	3	Leuctridae	1.86
Perlodidae	2.72		
Limnephilidae	2.7		
Hydropsychidae	2.58		

## Conclusion

Urban rivers face challenges which influence the diversity, abundance and distribution of their organisms and some of these changes are irreversible. The metrics used to assess the river including total number of organisms, biotic indices (the BMWP, ASPT and WHPT), physicochemical variables and Gammaridae-Asellidae ratio have shown the river could be in ‘good’ condition especially at the downstream locations. The application of WHPT helped to improve our understanding of abundance weighting applied for benthic macroinvertebrates in river classification. With higher percentage urban cover recorded at the lower sections, the river quality has been shown not be in a worst condition but demonstrated that urban influence could impact on the river’s biodiversity. Modifications of urban rivers are permanent; their management will therefore require treatment on a case by case basis in order to effectively manage the urban river as a sustainable resource for all. An integrated catchment approach could be adopted which includes river basin development groups, catchment caretakers, environmental regulators, water companies and members of the public to protect and sustain our rivers and other waterbodies. While some parts of the urban river will require maintenance, others will need to be restored and prevention of more urbanised areas will be a major challenge as human population and development increases.
